# The iTHRIV Commons: a cross-institution information and health research data sharing architecture and web application

**DOI:** 10.1093/jamia/ocab262

**Published:** 2021-11-25

**Authors:** Johanna Jean Loomba, Glenn S Wasson, Ravi Kiran Reddy Chamakuri, Pabitra Kumar Dash, Stephen G Patterson, Mary M A Potter, Jason Edward Krisch, Martha M Tenzer, Karen C Johnston, Don E Brown

**Affiliations:** 1 Integrated Translational Health Research Institute of Virginia, University of Virginia, Charlottesville, Virginia, USA; 2 UVA Health Analytics, University of Virginia, Charlottesville, Virginia, USA; 3 Research Computing, University of Virginia, Charlottesville, Virginia, USA; 4 Research & Clinical Trial Analytics, University of Virginia, Charlottesville, Virginia, USA; 5 Scholarly Integrity and Research Compliance, Virginia Tech, Blacksburg, Virginia, USA; 6 Fralin Biomedical Research Institute, Virginia Tech, Roanoke, Virginia, USA; 7 Health Analytics Research, Carilion Clinic, Roanoke, Virginia, USA; 8 Department of Neurology, Integrated Translational Health Research Institute of Virginia, University of Virginia, Charlottesville, Virginia, USA; 9 School of Data Science, Integrated Translational Health Research Institute of Virginia, University of Virginia, Charlottesville, Virginia, USA

**Keywords:** information storage and retrieval, patient-generated health data, computer systems, data commons, health research software

## Abstract

**Objective:**

The integrated Translational Health Research Institute of Virginia (iTHRIV) aims to develop an information architecture to support data workflows throughout the research lifecycle for cross-state teams of translational researchers.

**Materials and Methods:**

The iTHRIV Commons is a cross-state harmonized infrastructure supporting resource discovery, targeted consultations, and research data workflows. As the front end to the iTHRIV Commons, the iTHRIV Research Concierge Portal supports federated login, personalized views, and secure interactions with objects in the ITHRIV Commons federation. The canonical use-case for the iTHRIV Commons involves an authenticated user connected to their respective high-security institutional network, accessing the iTHRIV Research Concierge Portal web application on their browser, and interfacing with multi-component iTHRIV Commons Landing Services installed behind the firewall at each participating institution.

**Results:**

The iTHRIV Commons provides a technical framework, including both hardware and software resources located in the cloud and across partner institutions, that establishes standard representation of research objects, and applies local data governance rules to enable access to resources from a variety of stakeholders, both contributing and consuming.

**Discussion:**

The launch of the Commons API service at partner sites and the addition of a public view of nonrestricted objects will remove barriers to data access for cross-state research teams while supporting compliance and the secure use of data.

**Conclusions:**

The secure architecture, distributed APIs, and harmonized metadata of the iTHRIV Commons provide a methodology for compliant information and data sharing that can advance research productivity at Hub sites across the CTSA network.

## INTRODUCTION

The objective of the iTHRIV Commons is to provide a cross-site scalable research infrastructure that enables information sharing as well as the secure collection, storage, sharing, and analysis of data in accordance with the NIH endorsed FAIR (Findable, Accessible, Interoperable, and Reusable) principles.^1^ The system must meet administrative, security, legal, informatics, regulatory, and research requirements from a diverse set of institutions, while supporting both manual and automated collection of searchable metadata and providing a managed lifecycle for health research data. The product must allow research teams from different departments and schools to integrate their research workflows while providing personalized access as controlled by project owners and data administrators. The system should also support workflows for secure and well-governed transmission of medical record data to research teams, within and across the iTHRIV network. This article describes the design and early implementation of this product, demonstrating how to meet this broad set of requirements and how to harmonize research workflows and tools across institutions, supporting the sharing of some artifacts while still allowing for security and governance controls at the local level.

## BACKGROUND AND SIGNIFICANCE

The integrated Translational Health Research Institute of Virginia (iTHRIV) is a collaboration of public academic institutions (the University of Virginia and Virginia Tech) and private hospitals (Inova Health and Carilion Clinic) across the Commonwealth of Virginia that supports translational research. The mission of iTHRIV is to catalyze and sustain inclusive clinical and translational research through diverse, collaborative team science, innovative data science, and broad workforce development in order to improve human health and promote health equity.^2^

Navigating the complex health research landscape can be challenging for research teams, administrators, and service providers. Targeted communication is always difficult in research communities, but it became an even bigger challenge when iTHRIV’s multicenter Clinical and Translational Science Awards (CTSA) consortium opened the door to more cross-state resource sharing. Similarly, health data management was already a complex task at each institution, but cross-site workflows often meant a further reduction in efficiency. Previous system solutions for health data management at our iTHRIV institutions often involved project-driven, siloed technology and the appropriation of tools and systems not specifically designed for research workflows. With regard to medical record data, workflows that support patient care were often supplemented at the project or departmental level with custom databases or distributed for storage on secure servers managed by a variety of teams. This redundancy was resource intensive and prohibited findability and interoperability. Regulatory and policy requirements also posed a challenge for researchers who had to generate and manage compliant solutions based on the specific composition of their team and the data requirements of their project. Although health system policy and Institutional Review Board requirements set rules for the management of sensitive data, there was no streamlined and auditable system whereby health data stewards could transfer data sets to researchers. Generation of an appropriate data security plan, and subsequent compliance over the life of the project, typically required researchers to understand and oversee system requirements well-outside their areas of expertise. Investigators across our institutions had to spend significant time and financial resources to address these challenges.

Our research workforce needed a secure and compliant system to share information and data in order to facilitate collaborative research. Data commons have been shown to provide the framework to meet these research needs through the colocation of data, storage, and computing infrastructure.^3^^,^^4^ While these existing approaches to the data commons are providing solutions to important data sharing problems, they have been built for structured data and for specific focus areas, such as genomics.^5^ The NIH Data Commons pilot initiated in 2017 aimed to solve some of the well-recognized issues around data access and sharing, but that initiative was not extended beyond the initial pilot, leaving academic institutions to design their own solutions based on best-practices and lessons learned in the NIH pilot.^6^ Recent work has also shown that rather than a single information commons at the national level, a more effective approach to clinical translational research is a distributed set of connected “commonses.”^7^ As discussed below, this distributed model is the approach in the iTHRIV Commons with an architecture comprised multiple landing services hosted behind the firewalls of each institution.

In response to these common needs across our CTSA, and in the absence of an existing product meeting all requirements, we designed and developed a custom but cost-effective distributed platform for sharing information and securely managing data. This integrated translational research eco-system provides a cross-institutional index of information, resources, projects, and data sets. Unlike other approaches data commons, the scope of the iTHRIV Commons is broad, meaning that the product supports all types of translational health research. It also gives the appropriate entities the ability to expose some artifacts while protecting others. This ensures the proper stewardship and privacy protection that is essential to the translational research process. These protections enable societal trust in the researchers,^8^ as well as, conformance to ethical and legal standards.

## MATERIALS AND METHODS

**Figure 1.**
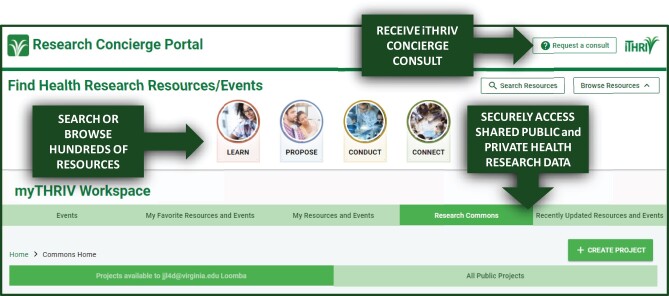
The dashboard of the iTHRIV Research Concierge Portal includes access to informational resources and events, concierge consultation services, and the iTHRIV Research Data Commons.

The iTHRIV Commons has been developed as a cross-institution ecosystem of tools, educational content, translational research events, and expert consults. As the primary entry point to the iTHRIV Commons, the iTHRIV Research Concierge Portal supports broad information and resource sharing using harmonized metadata, and now also provides secure federated access to locally stored data (Figure  1). Building and connecting the Research Data Commons to this web application required both technical innovation and careful consideration of data governance, security, and privacy requirements across the diverse participating iTHRIV partner sites. The features and system design of the Commons are explained in detail below.

### iTHRIV Commons features: expert consults

The iTHRIV Research Concierge Portal provides users with access to a range of CTSA-supported consulting services. Consult requests, tied to user-selected topics, are automatically routed to the appropriate team of experts based on the user’s home institution. These teams may be different for each institution (such as when the topic selected is “Medical Record Data Pull”) or might consist of a cross-CTSA team (such as when the topic selected is “Community Seed Grants”). Consult requests submitted through the iTHRIV Portal generate service management tickets which are tracked for purposes of satisfaction and resource-utilization metrics.

### iTHRIV Commons features: informational content

The iTHRIV Research Concierge Portal provides a single online entry point to essential clinical and translational research tools and resources, with customized views of content based on users’ institutional affiliation and object-level permissions.

The Portal provides templates for resources and events, with the aim of supporting team science, community engagement, and innovation in health-related research. Content is crowd-sourced; any user with an iTHRIV institutional login is able to quickly build new portal pages and submit to site administrators for curation and approval. Personalized features are also available to authenticated users, including a “favorites” menu, private views of draft content, and access to consultations service lines based on their associated institution.

All users can switch from a filtered view of resources and events curated for them to a broader view, allowing users to focus their browsing. The Portal also offers a limited-access public view, with content curated for the public. The many-to-many relational mapping between resources and categories ensures that users find all content related to their area of interest while browsing, while ensuring that content is not duplicated across the site (see Supplementary Table S1). An Elasticsearch index supports faceted searches of content in the iTHRIV Commons. User views are provided in Supplementary Figures S1–S5.

### iTHRIV Commons features: research data

In addition to information and resource management, the Portal also provides tools for project and data management. The foundational functionality of the iTHRIV Research Data Commons facilitates data access and compliance:

- Granular permissions are defined at the project and individual data set level. The Principle Investigator and delegated Project Owners manage the study team permissions and project metadata. Access to project data sets is independently controlled by Data Set Administrators, which may include study team members or stewards of research repositories, medical record systems, research labs, etc. Each project team member has a custom view and may only access the associated objects which they are permitted to see.
- Highly Sensitive Data (HSD) access is limited via an interface with the respective iTHRIV organization’s Institutional Review Board (IRB) database API. If a data set contains HSD, the data set admin must associate an active local IRB protocol with the data set prior to uploading any files. Data set administrators can only assign permissions to appropriate active study personnel as listed on the approved IRB protocol. If a researcher becomes inactive on the protocol in the IRB database, their access is automatically removed; if the protocol becomes inactive, data access is blocked.
- Access for research team members at partner institutions is systematically restricted to limited data sets or deidentified data and requires that a project-specific contract first be uploaded into the project.
- Integration with research databases, such as REDCap, allows periodic, scheduled extracts that may be shared with the appropriate project stakeholders. This allows project owners to expose certain elements of their data to a broader audience while maintaining control and protection over the source data.
- Manual and automated population of project and data set descriptions and characteristics (metadata) support data organization, discovery, and compliance. Permissions are independently controlled for project metadata, data set metadata, and data set files. This allows researchers to share descriptions of their work with the research community while having the option of keeping the actual data files hidden for most users.
- The system facilitates administrative reporting and auditing of research data assets. Audit logs reflect all access to both public and private objects in the Commons.

### iTHRIV Commons: metadata

Resources, events, projects, and data sets all have a well-defined set of metadata that supports discovery and enables the system to apply business logic based on object characteristics. Sensitive data is delineated at the field level in the metadata (ie, Street Address) and the system applies logic to set the value of hidden metadata fields that drive possible actions around that object (ie, sensitivityLevel = “HIGH SENSTIVITY DATA” can never be made public). Uploaded data files are scanned to check for accuracy of metadata. All data set uploads require the population of a core set of metadata with metadata extensions provided based on data set type as defined by the user. For example, if the user defines the data set as DICOM data, additional imaging metadata fields appear, most of which are auto-populated at the time of file upload using header information in the files. Schema.org metadata is used whenever possible to provide maximal findability to external search engines and also to enable greater interoperability with other data repositories^9^ and other large biological sciences metadata projects using this annotation.^10^ Comprehensive metadata documentation is provided in the Supplementary Materials.

### iTHRIV Commons: governance

**Figure 2.**
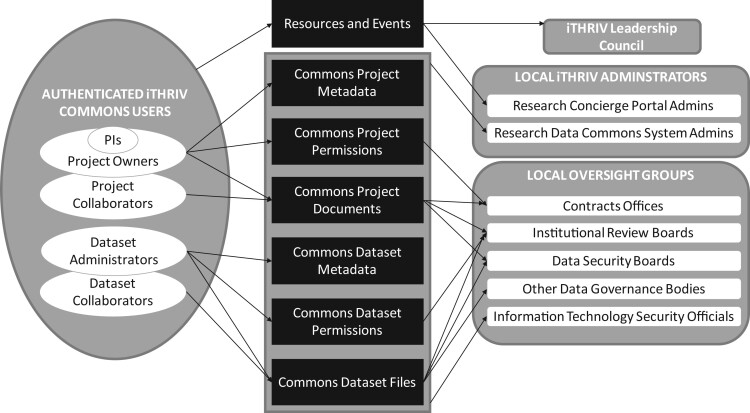
This diagram illustrates governance and oversight over various object types in the iTHRIV Commons. Note that the governance is largely managed at each local institution. Thus, the system provides the overall framework for governance while remaining agnostic to variations at the local level.

Proper governance, stewardship, and protection of data are paramount when managing potentially sensitive research data. System integration with local Institutional Review Boards (IRBs) ensures that work in the Commons aligns with federal regulations pertaining to ethical human subject research^11^ without introducing redundancy with existing IRB processes and systems. Similarly, existing legal and security standards are integrated into the system whenever possible to ensure local governance bodies continue to play their appropriate roles while still providing the researcher with a shared single system for data management. Figure  2 illustrates which users control various objects types in the iTHRIV Commons as well as the governing bodies providing oversight and Table  1 provides further details. User roles and object access flags related to content ownership and governance are provided in the Supplementary Materials.

**Table 1.** This table delineates ownership, governance, and oversight for each object type in the iTHRIV Commons

Object type	Object ownership	Object governance and oversight
**INFORMATIONAL RESOURCES AND EDUCATIONAL EVENTS**	**Authenticated iTHRIV Commons user** listed as an owner in object metadata	Any **authenticated iTHRIV Commons user** can add new draft resources and events. They may access and edit content along with other owners they assign. Final content requires approval by local the **iTHRIV Research Concierge Portal Administrators** before becoming visible to other user groups. Portal admins are designated by each site prior to local launch and follow locally established processes for their review and approval of content. The **iTHRIV Leadership Council** provides overall guidance on appropriate range of content, but general scope is anything that supports the translational health research process throughout its lifecycle.
**RESEARCH PROJECT METADATA** (descriptions and attributes)	**Principal Investigators** and other users they designate as **Project Owners**	**Principal Investigators** govern their projects and decide whether or not to make the metadata discoverable for all users via the iTHRIV Commons index. As metadata is stored locally at each institution, local **iTHRIV Research Data Common System Administrators** can block metadata access to one or all projects at their institution.
**RESEARCH PROJECT PERMISSIONS**	**Principal Investigators** and other people they designate as **Project Owners**	**Principal Investigators** govern who has access to their private projects and the content therein. **Users from Partner Institutions: Institutional Contracts Officials** govern terms surrounding cross-institution collaborations. The Project Owner can only add permissions for users from partner institutions once an executed contract has been uploaded to the project documents. Note that **Security Officers** at each institution identify secure IP address ranges that are permitted to access projects in the private Research Data Commons. Permissions are stored locally and each institution’s **iTHRIV Research Data Common System Administrators** can remove permissions at any time.
**RESEARCH PROJECT DOCUMENTS** (protocols, contracts, data security plans, publications, etc.)	**Project Owners** and **Project Collaborators**	Although the research team manages the documents they upload, the system requires that certain documents be present in order to unlock features that require additional governance (as described in other rows of this table).
**RESEARCH DATA METADATA** (descriptions and attributes)	**Data Set Administrators**	A single project may use data sets that are stewarded by individuals within or outside the project team. The **Data Set Administrator** could be the electronic health record steward, biorepository manager, lab manager, or anyone responsible for controlling access to the source data. The data set admin may be the **Principal Investigator** if novel data is prospectively generated or collected through the course of the project. The responsible data admin creates a data set object within a project and defines the metadata attributes. They also decide whether or not to make the metadata discoverable for all users via the iTHRIV Commons index. **HIGHLY SENSITIVE DATA (HSD) CONTAINING PHI:** Automated scans help ensure that files potentially containing any HIPAA data element that has not been explicitly defined in the metadata are flagged for review by data set admin and an email is sent to local **iTHRIV Research Data Common System Administrators** to prompt audit.
**RESEARCH DATA SET PERMISSIONS**	**Data Set Administrators** and **Institutional Review Boards**	A Data Set Admin must first be added to Project by a Project Owner, but once added they independently control access to their respective data sets. They can assign access to a subset of the project team. **HIGHLY SENSITIVE DATA (HSD) CONTAINING PHI:** Local **Institutional Review Boards (IRBs)** govern access to HSD via API integration with their systems. The iTHRIV Commons only allows the data set admin to grant permissions to researcher who are approved by the IRB for the associated study protocol. Permissions are automatically deleted if investigators are removed from the study protocol in the IRB system. **iTHRIV Research Data Common System Administrators** can remove permissions at any time.
**RESEARCH DATA FILES**	**Data Set Administrators** and **Data Set Collaborators**	Data file uploads and downloads are done by data set admins and collaborators with change history preserved for audit and prior versions remaining available to the study team. **HIGHLY SENSITIVE DATA (HSD) CONTAINING PHI:** HSD is governed by the local **Institutional Review Board**, so the system automatically freezes access to these objects if the associated study protocol becomes inactive. At the time of download, users are presented with the associated data security plan (approved by local **Data Security Offices**) and must agree to download to an approved location prior to proceeding. Audit logs reflect user activities. **PUBLIC DATA SETS:** Governance rules, guidelines, and processes for publishing data sets are established by the respective **Institutional Data Governance Authorities** and incorporated into the automated workflow. At a bare minimum, the system restricts publishing data sets containing any sensitive and/or personally identifying information. **iTHRIV Research Data Common System Administrators** can freeze access to the data sets at their own institution any time issues are identified on audit or if otherwise indicated by governing bodies.

### iTHRIV Commons: review of compensating controls

Multiple approaches are taken in the iTHRIV Commons to minimize risks (data misuse and noncompliance).

- Policy Considerations: Implementation of the Commons involves a careful review of data protection standards, records management practices, and research team training requirements at each site. System features are configurable at the site level if needed.
- System Security: Firewall protections (F5 and VPN), Apache/Linux Hardening, and an Open Web Application Security Project (OWASP) Top 10 Web Application Firewall^12^ contribute to the security of the system. Regular monitoring by auditors and institutional security teams assures these measures are sufficient.
- Regulatory Considerations: IRB database integration ensures that regulation around access to highly sensitive data is carried over from the IRB application process into real-time data management practices in the Commons.
- User Accountability: Commons users must sign an end-user agreement upon entering the Commons (and at time of agreement update). Users maintain responsibility for their own actions pertaining to data management as trained by their local institutions, but the system supports compliance with built in business logic and user prompts. For example, users must attest to having reviewed the associated data security (provided as a linked document) prior to downloading any sensitive data.
- Account Management: iTHRIV institutions retain local control over the management of provisioning and deprovisioning user accounts. Integration with LDAP systems ensures continual alignment with local policy as well as supporting timely deprovisioning of access to Commons objects upon departure of faculty and staff.
- Legal Implications: The Commons software is deployed under contractual agreements (described below).

### iTHRIV Commons: contractual considerations

The iTHRIV Master Agreement describes the NIH-NCATS supported partnership between the University of Virginia (UVA), Inova Health, Carilion Clinic, and Virginia Tech. This agreement supports the shared access to resources and events in the iTHRIV Portal. An addendum to this agreement describes the legal responsibilities of these parties with respect to the federated iTHRIV Research Data Commons feature. Execution of this addendum is required prior to installation of the iTHRIV Commons Landing Service at partner institutions and prior to any cross-site access to Research Data Commons objects. Projects involving data sharing across partner sites still may have unique legal considerations around data provenance, intellectual property, or other factors. Thus, cross-site data sharing still requires that an executed contract be uploaded to the Commons project prior to adding collaborators from partner sites.

### iTHRIV Commons: research concierge portal architecture

**Figure 3.**
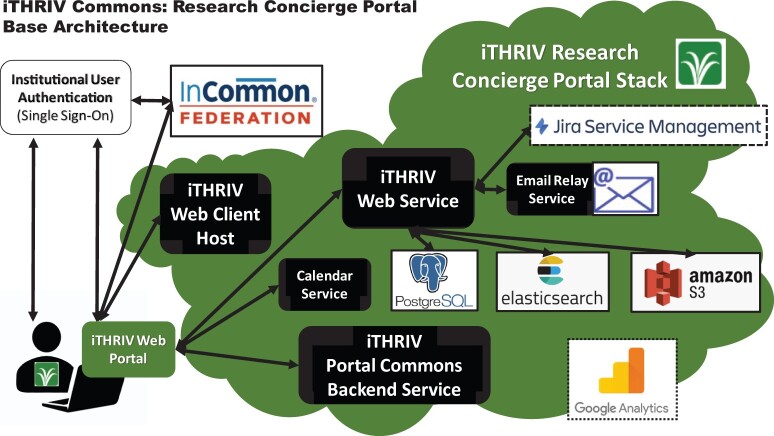
The iTHRIV Research Concierge Portal is a complex full-stack cloud-hosted web-application shared by all partner sites with a limited view and a sub-set of services provided to the public. The component relationships are illustrated here.

The iTHRIV Research Concierge Portal is a custom-built full-stack web application, currently hosted in Amazon Web Services (AWS). Front-end code, run on the client web browser, is written in Angular. User authentication is done through respective institutional identity management systems via the Internet2 InCommon Federation,^13^ the signer and curator of US research and education trust registry information used in federated transactions globally. Reliance on institutional logins ensures that system access remains aligned with each institution’s user account access (such as dual-authentication) and password requirements as they change over time. Each identity provider issues a browser token for the session which is stored in the client’s browser while session information is passed back to the iTHRIV Portal Backend Service in AWS. Users can also bi-pass authenticated login by selecting the “PUBLIC” icon on the login screen. The user’s browser connects to https://portal.ithriv.org/#/home and runs the iTHRIV Portal Client code; content on the user’s web client is then dynamically rendered via RESTful API calls to the iTHRIV Web Service. Figure  3 shows the interactions between the user’s machine, the systems involved in authentication, and the components of the iTHRIV Research Concierge Portal, with detailed descriptions of the latter provided in Table  2.

**Table 2.** The iTHRIV Research Concierge Portal full-stack components

iTHRIV research concierge portal system components	
AWS instance	An image of Center for Internet Security (CIS) Ubuntu Linux 18.04 in AWS hosts the iTHRIV Research Concierge Portal.
iTHRIV web client host	Provides the client with code to run on the browser, dynamically rendering page content when API calls to the various services are executed.
iTHRIV web service	A RESTful API service that receives requests from code running on the client and returns content from the Portal backend.
iTHRIV portal commons backend service	Middleware (python flask application) that manages all metadata interactions between the client and the iTHRIV Commons Landing Services APIs, hosted at respective iTHRIV institutions. [Available to authenticated users only.]
PostgreSQL database	Resource and Event object metadata are stored in this database.
Elasticsearch	Supports indexed search of objects stored in the PostgreSQL database.
Amazon S3 Bucket	Provides Storage for files (including audio and video files) that are attached to Resource and Event objects in the portal.
Jira service management	A licensed service management software that the iTHRIV Web Service integrates with via backend APIs. User consult requests in the Portal generate a ticket in one of more than 20 different iTHRIV Jira projects depending on the user’s home institution and request type. Service teams manage the tickets through the Jira software, but users can access and track the status of their tickets at any time in the iTHRIV portal interface. [Available to authenticated users only.]
Calendar service	This plug-in supports event features in the Portal, allowing the user to select dates and add events to their personal calendars.
Email service	An email relay service handles the sending of system emails, such as requests for resource page approval by iTHRIV admins. [Available to authenticated users only.]
Google analytics	Provides iTHRIV with web application metrics.

### iTHRIV Commons: expanded system architecture for Research Data Commons feature

**Figure 4.**
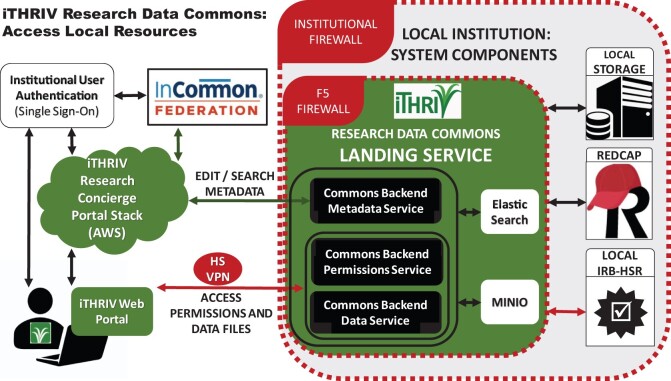
This diagram is a simple representation of the system interactions between an iTHRIV researcher’s computer, the iTHRIV Research Concierge Portal Web Application (collapsed here but with details available in Figure  3), ancillary authentication services, and the iTHRIV Research Data Commons Landing Service at a single site. Hardware and operating system support are provided by each participating institution, including firewalls, file storage, and servers that support file storage and the Landing Service software components.

Implementation of the first phase of iTHRIV Research Concierge Portal (informational content only), involved the full stack web-application which was described above. Implementation of the iTHRIV Research Data Commons feature required adding an iTHRIV Commons Adapter Service (Python Flask application) to the iTHRIV Research Concierge Portal backend as well as the installation of a multi-component iTHRIV Commons Landing Service behind each institutional firewall (Figure  4 and Table  3). Note that the user must be on high-security VPN or on a secure local area network in order to access the Commons Landing Services’ APIs. (This is not a requirement for accessing other features in the iTHRIV Research Concierge Portal.) Each landing service is configurable for integration with local research management systems, including IRB databases, research databases, and storage. The flexible framework supports adaptation for each institution to its own systems, processes, and policies.

**Table 3.** The components of a local instance of an iTHRIV Research Data Commons Landing Service, including services and software deployed by the iTHRIV development team and existing local system that are integrated into the product

iTHRIV research data commons: system components at participating institutions	
Firewalls	Institutional firewalls are maintained according to local standards. An F5 appliance sits between the external (AWS and client browser) components and internal system components (institutional iTHRIV Commons Landing Service and local research system assets), and provides an additional overlay of Application Security Services. For example, F5 ensures that Landing Service API calls can only be made from the Portal or from IP addresses associated with approved IP ranges from iTHRIV partner sites.
iTHRIV Commons Landing Service APIs	Three custom Python Flask web applications, deployed by the central iTHRIV development team and installed behind each local firewall: Commons Backend Metadata Service: Provides APIs for metadata CRUD. Commons Backend Data Service: Provides APIs for data file upload/download/delete operations. Commons Backend Permissions Service: Provides APIs for managing user access permissions on projects and data sets.
MinIO software	Object store for storing/retrieving data files uploaded by users as well as storing a copy of project/data set metadata. Metadata is saved here to make it possible to rebuild Elasticsearch metadata index as well support metadata versioning feature.
Elasticsearch software	Provides service for managing project and data set metadata and facilitates search.
Local IRB databases	The Landing Service APIs make calls to the local IRB Database APIs to retrieve protocols and study team information, supporting alignment between Commons permissions and IRB approvals for use of HSD. This integration ensures that the
Local research databases	REDCap integration allows researchers to request routine exports of from their existing research databases to the Commons where they control permissions for further sharing of their data. Each new export request is reviewed by local REDCap teams for regulatory compliance before the providing the landing service with the token for scheduled routine extract.
Local storage	Data set metadata can point to other ancillary storage systems, allowing for files to remain in their current location while being indexed and managed in the Commons.

**Figure 5.**
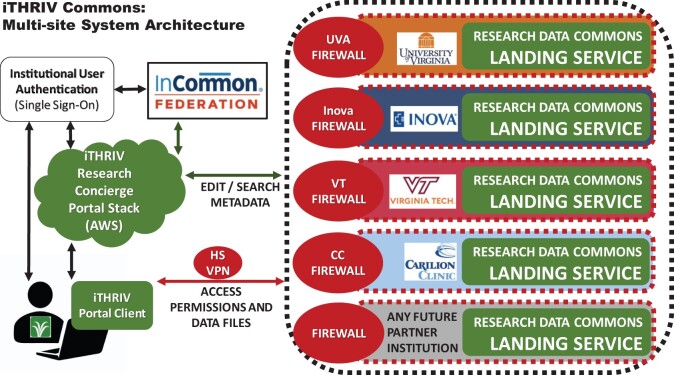
This diagram is a simple representation of the federated iTHRIV Research Data Commons, with an iTHRIV Research Data Commons Landing Service installed at each site. The current iTHRIV Partner institutions are listed here, but other institutions may join the federation in the future under appropriate product licensure. To see the full system components involved at each participating site labeled in this diagram, refer to Figure  3. To see the full iTHRIV Research Concierge Portal Stack components, refer to Figure  2.

As noted above, an iTHRIV Commons Landing Service is installed at each site (Figure  4). The user will access Commons objects (according to their permissions) across all the partner sites via API calls across the federation of landing services (Figure  5). Metadata is sent back via the Portal and then sent to the client’s browser. In contrast, API calls related to object permissions or the uploading or downloading of data files are sent directly from the client (connected to a secure network) and through F5 and the local firewall to the local landing service. Thus, sensitive information is *never* routed through the iTHRIV Research Concierge Portal Backend Service in AWS.

## RESULTS

### iTHRIV Commons: research concierge portal launch

The iTHRIV Research Concierge Portal was launched at the University of Virginia in 2018 with a base set of features supporting crowd-sourced resource sharing. Over the next 2 years, the portal was launched at all partner iTHRIV institutions and new features were added, including the ability to add and manage translational research-related events and an integrated concierge consultation service. With >10 000 users per Google Analytics, 829 resources, 325 events, and >1500 consult requests to date, this cross-institution web application has provided iTHRIV institutions with a foundational design for the federation of information and researcher support.

With its curated and personalized views, the platform has proven invaluable for the launch of programs that are developed at a single institution, then expanded and generalized for use across iTHRIV partner sites and eventually shared with the CTSA consortium. One example is the iTHRIV Research Administration Portal for Training and Resources (RAPTR) program which has been constructed as a set of interconnected iTHRIV Research Concierge Portal resource pages.^14^ The portal enables RAPTR creators to curate each training resource page based on the audiences for which it is intended by defining “Institutions with Access” in the metadata of each page. Users from each institution are presented with customized training around local processes as they navigate, while users from outside institutions will be presented with generic templates that can be adapted to fit their local research administration processes. The broader resource sharing supports efficiency and standardization across institutions while the local views support more targeted content, all within a single platform.

Resource content is as varied as the landscape it reflects, but the evolution and use of the portal has demonstrated how metadata can be harnessed to focus the users’ experiences even as the breadth of available resources is expanding. Analytics show us that users both browse categorically and apply filters in faceted searches to discover content. The content is associated by its owner with one or more subcategories (Supplementary Table S1) and assigned one primary “type” (Table  4). The values for these object metadata were defined by a collective team of iTHRIV Portal Admins with representatives from each site and have been dynamically refined over time via an administrator interface based on user feedback and domain developments, such as when a “COVID Collaboration Corner” was added in April of 2020.

**Table 4.** This table provides current counts of resource content in the portal by “type,” a system defined set of 10 tags that are more generic than the 95 subcategories but that provide another method for refining a search

Resource type	Current count
Education	396
Other research resource	158
Informatics/analytics	148
Funding resource	89
Regulatory and compliance	88
Center or initiative	82
Administration	59
Research cores and labs	58
Learning shot	44
Health system	22

*Note:* Each resource is assigned only one type with “Other Research Resource” being used for content that does not belong to the other specific types.

### iTHRIV Commons: research data commons launch

The first iTHRIV Commons Landing Service has been installed at the University of Virginia (UVA) and integrates with local storage endpoints as well as the local IRB-HSR database. The product was initially deployed in User Acceptance Testing (UAT) environments for feature acceptance by the product owner then testing by the development team, the health data analytics team, and iTHRIV staff. This round of testing provided a valuable debugging opportunity and led to usability enhancements and planned future feature development.

At the time of launch, this landing service is only accessible to authenticated UVA users who are connected to a high-security UVA network (via LAN or VPN connection). iTHRIV has initiated a phased roll-out of Version 1 of this product at UVA; the research teams invited to use the first production version of the product were selected for breadth and depth of their projects. Their cross-department teams may typically face stewardship challenges when sharing their data. Their complex projects also benefit from advanced features in the Research Data Commons such as auto-population of imaging metadata and integration with REDCap.

Pending institutional review of system performance in this pilot phase, iTHRIV plans to expand use of the system to all UVA researchers conducting clinical and translational research later this year. iTHRIV partner institutions (Virginia Tech, Inova Health, and Carilion Clinic) will be installing an iTHRIV Commons Landing Service and integrating it with their local research data environment. This will support management of local private projects and data sets, object sharing across iTHRIV when applicable, and the publishing of research artifacts from partner site research.

**Figure 6.**
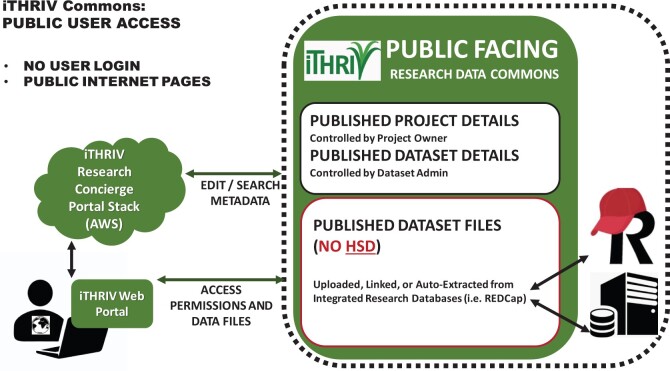
A simple diagram representing planned public access to the iTHRIV Research Data Commons. Note that no authentication is required.

A public view of shared informational content is already supported in the iTHRIV Portal. In 2022, iTHRIV expects to support public access to approved project and data set metadata as well as published data sets (Figure  6). Public data access will always be mitigated by integrated IRB controls, institutional governance policies, and will also require approval by project owner and data set admin. Thus, researchers will retain the right to not expose metadata or files related to their private projects.

## DISCUSSION

The information sharing supported by iTHRIV Research Concierge Portal, in use by all 4 iTHRIV institutions and public users, has established a foundational translational research infrastructure that opens doors to further feature enhancements and cross-institutional data sharing.

### Product customization and feature enhancement

With additional institutional and project funding, the iTHRIV Research Data Commons software will continue to be extended to meet the needs of specific research projects. In the past, projects developed custom, siloed solutions. In contrast, the Commons provides a robust foundation and a ready-made set of data management tools upon which new features can be built (ie, new system integrations, metadata extensions, analytics pipelines, etc.). Sharable software (analytics tools) will be added as the next object type in the Commons with a corresponding set of metadata and business rules.

Developing custom tools as an extension of the iTHRIV Commons will ensure new data management tools and solutions are integrated into this cross-institution platform. Thus, project-specific data management innovations can result in system enhancements that will benefit all future researchers across iTHRIV as well as our community partners. This shared resource will expose a growing index of projects and data sets, while visibility and access continue to be controlled at a granular level. The expansion of the iTHRIV Research Data Commons can support any number of new and developing research domains, thereby creating an economy of scale and also supporting organic interdisciplinary interoperability and cross-pollination.

### Potential for future product dissemination

The iTHRIV Commons has been designed to that it can be deployed in the future at any institution that supports login via the InCommon federation and that is capable of installing the landing service APIs, Elasticsearch, and MinIO and doing some local configuration (assigning local system admins, integrating with local storage endpoints, making API calls to local IRB). The IRB API calls are agnostic to vendor as long as the basic information can be returned (active studies and active researchers on those studies). iTHRIV intends to license this product at no cost to other nonprofit translational research institutions.

## CONCLUSION

The iTHRIV Commons has fundamentally changed the piecemeal and segregated project approach to translational research within and among the iTHRIV partners. iTHRIV Portal usage statistics demonstrate that diverse teams across our iTHRIV institutions can now work in a single integrated environment, personalized for each user. The initial installation of the iTHRIV Research Data Commons Landing Service at UVA establishes the feasibility of implementing a distributed data management infrastructure.

Commons metadata supports regulated object-level permissions and provides an opportunity for systematic characterization, indexing, discovery, and auditing of institutional research data. We expect that the successful deployment of the iTHRIV Research Data Commons at partner sites will demonstrate the potential for further expansion of the Commons infrastructure to other external partners across the state as well as to other translational research institutions who wish to adopt this technology.

## FUNDING

This work was supported by National Center for Advancing Translational Sciences grant number UL1TR003015.

## AUTHOR CONTRIBUTIONS

The products described in the paper were initially conceived of by DEB and KCJ (iTHRIV Principal Investigators) and detailed throughout a series of iTHRIV Informatics meetings involving all authors. GSW, JJL, RKRC, PKD, and SGP were responsible for software design and development at the University of Virginia. MMAP, JEK, and MMT ensured that the product aligned with research and informatics processes and requirements at their local institutions. JJL drafted the initial manuscript under guidance from DEB and KCJ with section content contributed by PKD and RKRC. All authors reviewed and revised the manuscript, approved the final version to be published, and agree to be accountable for all aspects of this work.

## SUPPLEMENTARY MATERIAL

Supplementary material is available at *Journal of the American Medical Informatics Association* online.

## Supplementary Material

ocab262_Supplementary_Data
