# Supplementary material for: The iTHRIV Commons: a cross-institution information and health research data sharing architecture and web application
Source: J Am Med Inform Assoc. 2021 Nov 25;29(4):631–42. doi: 10.1093/jamia/ocab262 (PMC8922196; doi:10.1093/jamia/ocab262)
Supplement: ocab262_Supplementary_Data [file ocab262_supplementary_data.docx]

iTHRIV COMMONS RESOURCES and EVENTS METADATA VALUES: MODES, CATEGORIES, and SUB-CATEGORIES

| **LEARN** | **PROPOSE** | **CONDUCT** | **CONNECT** |
| --- | --- | --- | --- |
| **INTRO to TRANSLATIONAL RESEARCH**   - Definitions - Research Discovery Tools - Sample Research | [**FUNDING RESOURCES**](https://portal.ithriv.org/#/network/1012)   - [Federal and State Funding](https://portal.ithriv.org/#/category/121) - [Institutional Funds](https://portal.ithriv.org/#/category/124) - [Seed Grants](https://portal.ithriv.org/#/category/123) - [Private Funds](https://portal.ithriv.org/#/category/126) - [Community Grants](https://portal.ithriv.org/#/category/122) | **ADMINISTRATIVE TOOLS**  - Clinical Research Management Systems - EPIC Research Features - Financial Management | **RESEARCHER CONNECTION**  - COVID Collaboration Corner - Community Resources for Researchers - Find Research Collaborators - Job Opportunities |
| [**TRAINING RESOURCES**](https://portal.ithriv.org/#/network/1008)   - [Research Admins](https://portal.ithriv.org/#/category/1041) - [Research Coordinators](https://portal.ithriv.org/#/category/32) - [Investigators](https://portal.ithriv.org/#/category/33) - [Community](https://portal.ithriv.org/#/category/31) | **BUDGETS, GRANTS and CONTRACTS**  - Budget Creation - Funded Agreements - Non-Funded Agreements - Grant Proposal Development | **LOGISTICAL RESOURCES**  - Investigational Drug Services - Tissue Management - Records Management - Study Participant Resources | [**COMMUNITY CONNECTION**](https://portal.ithriv.org/#/network/1022)   - [Clinical Research Access](https://portal.ithriv.org/#/category/304) - [Health Resources for Patients](https://portal.ithriv.org/#/category/305) - [iTHRIV Community Groups and Initiatives](https://portal.ithriv.org/#/category/302) - [iTHRIV Community Partners](https://portal.ithriv.org/#/category/301) |
| [**DATA SCIENCE and ANALYTICS**](https://portal.ithriv.org/#/network/1006)   - [Data Science](https://portal.ithriv.org/#/category/11) - [Biostatistics](https://portal.ithriv.org/#/category/12) - [Courses and Degrees](https://portal.ithriv.org/#/category/15) - [Data Visualization](https://portal.ithriv.org/#/category/13) - Available Datasets | **RESEARCH DESIGN**  - Feasibility - Experimental and Clinical Trial Design - Statistical Methods - Epidemiology - Survey Design | [**DATA MANAGEMENT**](https://portal.ithriv.org/#/network/1016)   - [Data Sources](https://portal.ithriv.org/#/category/201) - [Data Capture](https://portal.ithriv.org/#/category/202) - [Data Storage](https://portal.ithriv.org/#/category/203) - [Data and Safety Monitoring](https://portal.ithriv.org/#/category/206) - [Data Transfer](https://portal.ithriv.org/#/category/205) - [Data Analysis](https://portal.ithriv.org/#/category/204) | [**FIND OR DISSEMINATE RESULTS**](https://portal.ithriv.org/#/network/1024)   - [Find Research Results](https://portal.ithriv.org/#/category/1026) - [Publication Tips and Tools](https://portal.ithriv.org/#/category/333) - [Protection of Intellectual Property](https://portal.ithriv.org/#/category/331) - [Commercialization](https://portal.ithriv.org/#/category/334) - [Open Science](https://portal.ithriv.org/#/category/332) |
| **RESEARCH REGULATIONS**  - Basic Science Research - Animal Research - Human Subjects Research - Regulatory Bodies - Data Security | **WRITING ASSISTANCE**  - FDA IND/IDE Preparation - Grant Proposal Writing - Protocol Writing | [**REGULATORY MANAGEMENT**](https://portal.ithriv.org/#/network/1019)   - [Animal Research](https://portal.ithriv.org/#/category/232) - [Basic Science Research](https://portal.ithriv.org/#/category/231) - [Human Subject Clinical Research](https://portal.ithriv.org/#/category/234) - [Social and Behavioral Research](https://portal.ithriv.org/#/category/233) | [**RESEARCH CENTERS AND INITIATIVES**](https://portal.ithriv.org/#/network/1023)   - [Basic Science](https://portal.ithriv.org/#/category/323) - [Clinically Focused](https://portal.ithriv.org/#/category/322) - [Engineering and Technology](https://portal.ithriv.org/#/category/321) - [Public Health and Policy](https://portal.ithriv.org/#/category/324) - [General Translational Research](https://portal.ithriv.org/#/category/325) |
| **SPECIAL TOPICS**  - Community Engaged Research - Intellectual Property and Commercialization - Rigor and Reproducibility - Team Science - Equity | **REGULATORY REQUIREMENTS**  - Animal Research - Basic Science Research - Clinical Research - Social and Behavioral Research | **FACILITIES and CORES**  - Animal Research Facilities - Clinical Research Facilities - General Use Facilities - Health System Facilities - Research Cores and Laboratories | [**TRANSLATIONAL HEALTH RESEARCH ADMIN**](https://portal.ithriv.org/#/network/1025)   - [Federal Offices](https://portal.ithriv.org/#/category/345) - [NCATS CTSA resources](https://portal.ithriv.org/#/category/344) - [Offices of Research](https://portal.ithriv.org/#/category/341) |
|  | [**SPECIAL TOPICS**](https://portal.ithriv.org/#/network/1015)   - [Ancillary Services](https://portal.ithriv.org/#/category/167) - [Big Data Analytics](https://portal.ithriv.org/#/category/166) - [Ethical Issues](https://portal.ithriv.org/#/category/164) - [Intellectual Property](https://portal.ithriv.org/#/category/162) - [Multi-Center Studies](https://portal.ithriv.org/#/category/161) - [Subject Recruitment](https://portal.ithriv.org/#/category/163) | **SPECIAL TOPICS**  - Drug and Device Accountability - Ethical Issues - Recruitment Support |  |

*Supplemental Table 1: The iTHRIV Research Concierge Portal interface provides a refined hierarchical browsing experience. Note that content can be associated with more than one of the above subcategories, supporting discovery within a variety of contexts and workflows.*

iTHRIV COMMONS SAMPLE USER INTERFACE VIEWS


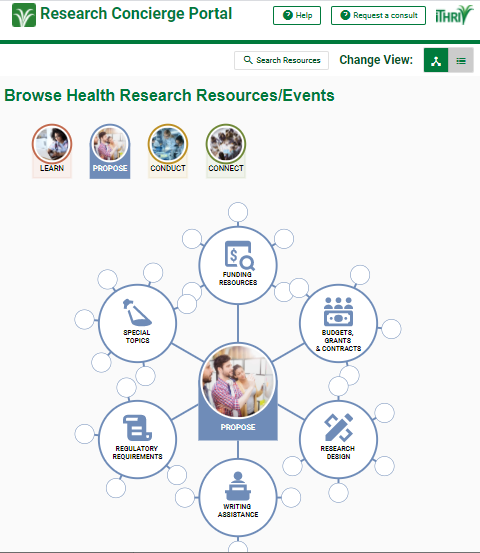


*Supplemental Figure 1: A user’s browsing view of the Propose mode categories.*


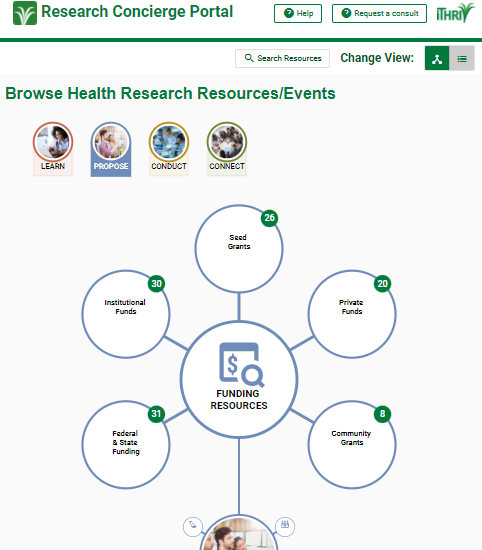


*Supplemental Figure 2: A user’s view of the subcategories in the Propose mode under the Funding Resources category.*


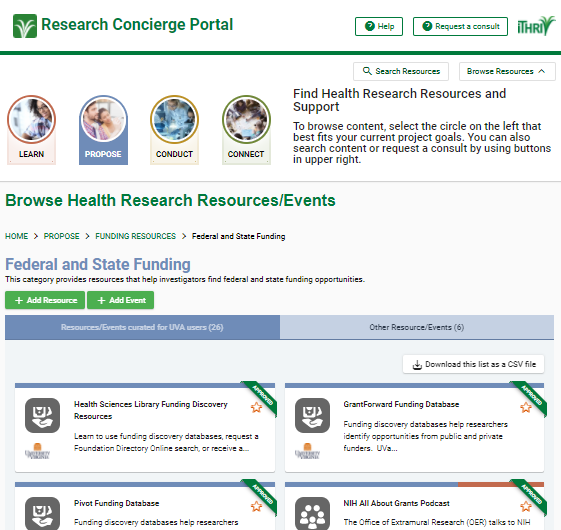


*Supplemental Figure 3: A UVA user’s view of some of the content in the Seed Grant sub-category. Content is ranked based on most favorited by users. The first tab displays content curated for users at my institution, but the second tab allows me to explore any non-private content curated for other users.*


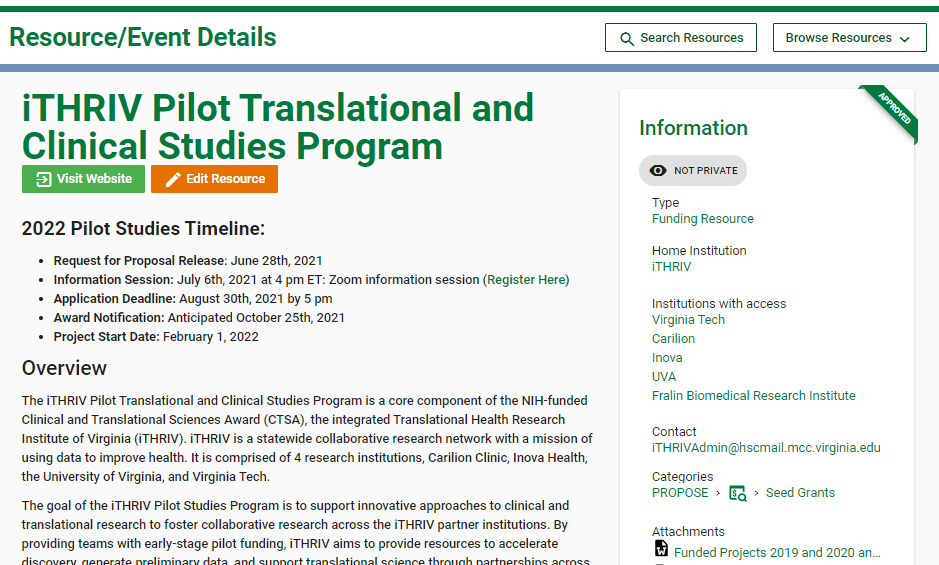


*Supplemental figure 4: A user’s view of a single resource object, with metadata visible in the pane to the right. Because the user is an owner of the page, they have the ability to edit the content.*


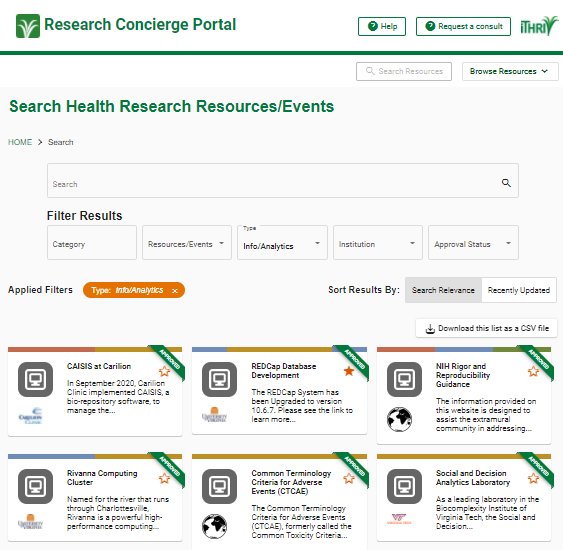


*Supplemental Figure 5: A user’s view of the faceted search after applying filter of object type “Informatics/Analytics”. Results (149 associated with this search) are paginated and can be further filtered by home institution, category, and approval status (available to local portal admins only).*

# iTHRIV Research Concierge Portal Statistics [as of Sept, 2021]

**
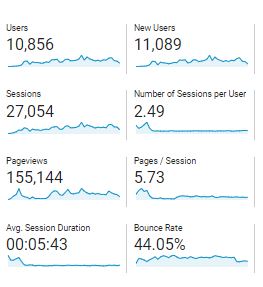
**


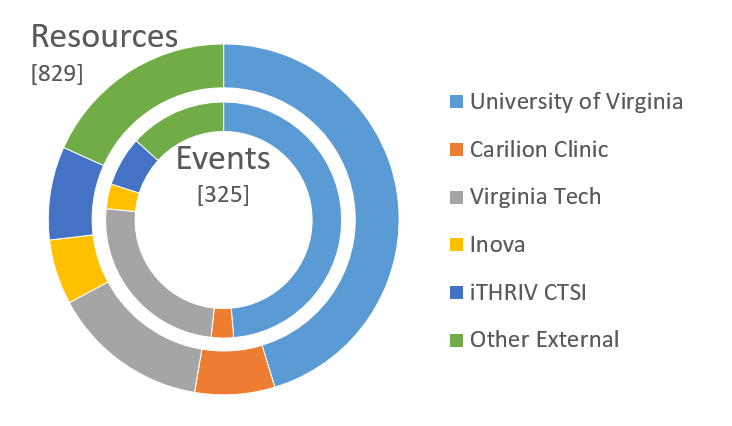


 iTHRIV COMMONS USER ROLES

1.       PUBLIC USER:

 Any user who is not an authenticated user.  They are accessing iTHRIV resources via a web client without any log in.  A basic via of the iTHRIV Research Concierge Portal is available to public users.  In the future, they will also have a view of all public metadata and datasets in the Research Commons.

 2.        iTHRIV MEMBER USER:

Any user who is a member of any of the iTHRIV participating institutions. UVA is their home institution or they belong to another iTHRIV institution that has signed the iTHRIV Master Agreement contract addendum for the Research Data Commons.  Users are authenticated by their respective iTHRIV institution when logging into the iTHRIV Portal.

3.       LOCAL SYSTEM ADMIN:

Each landing service has a small number of system admins who have back-end access to the APIs.  These admins can also take on an explicit Dataset Admin role when functioning as data stewards for the institution.  This is described below.  Note that in order to ensure maximum security, these permissions are hard coded on the backend in the landing service code, and not controlled via the APIs or interface.

4.       PROJECT OWNER:

The iTHRIV Member User who creates a project in the system becomes the initial project owner. A project owner has full project permissions including and exceeding project collaborators. A project owner can add other users to the project as owners. A project owner can add any member user as collaborator and can also add group iTHRIVCommonsAdmins@[institution email domain] as a collaborator.  A project owner can edit project metadata and set the metadata access flag (private/public). They can also delete any project they own. A project must have at least one owner. Any project owner can remove other project owners from the project. Any project owner can remove any project collaborator provided the collaborator is not the only dataset admin for a dataset of the project.  The project owner only has access to datasets attached to the project IF the respective dataset admins grant them that access. Project owners have all project collaborator permissions such as adding datasets and project documents.

 5.       PROJECT PRINCIPAL INVESTIGATOR (PI):

One or more people can be listed as the PI.  This is a role that might be meaningful for the project in terms of regulatory responsibilities, grant funds, etc.  However, from a permissions point of view, the PI is just another project owner.  It is possible they are the only project owner.

6.       PROJECT COLLABORATOR:

A user who Project Owner has added as collaborator.  When needed, landing service administrators can be added as collaborators by adding iTHRIVCommonsAdmins@[institution email domain] in the user id field. Project collaborators can add datasets to the project, manage project documents, and add to the project chat. A project can have collaborators from different iTHRIV institutions provided there is a contract document in the project. The project collaborator only has access to datasets attached to the project IF the respective dataset admins grant them that access.  When a collaborator adds a dataset he/she becomes the initial dataset administrator.

7.       DATASET ADMINISTRATOR:

When project owner or collaborator creates a dataset he/she becomes the default dataset admin for that dataset.  They are basically responsible for managing dataset for a project with full permission on the dataset. They can delete any datasets for which they have admin role. A dataset admin of a dataset can add other project team members as dataset admin for that dataset. They are also responsible for managing metadata for the dataset. A dataset admin can remove other admins from the dataset over which he/she has admin role. Thus dataset administrators are always a subset of project team members.  The iTHRIVCommonsAdmins@[institution email domain] may be a data steward for highly sensitive datasets.  These local system admins will have control over the metadata for these datasets and can upload dataset files for an approved IRB protocol research team.

 8.       DATASET COLLABORATOR:

A user who is given permission by the Dataset Administrator to work with (download/edit) the dataset file.  Note that dataset collaborators can see the file change history and can access previous versions of the dataset.  The dataset collaborator must first be listed on the parent project where the dataset lives in.  Thus dataset collaborators are always a subset of project team members.  In the case of highly sensitive data, these group is restricted and must be a subset of users associated with the approved IRB protocol.

9.       DATASET CUSTOMER:

Any iTHRIV Member User who is given explicit personal permission to download the current version of the dataset.  A customer does NOT have to be a project team member.  They cannot make changes (edits) to the file and they cannot upload new versions.  They cannot access older versions.  This is just personalized download privileges.

10.    DATASET CUSTOMER GROUP:

Each iTHRIV participating institution is considered as a group.  Entire groups can be granted dataset customer privileges (meaning a dataset admin could permit all users at a specific institution to download the current dataset).  This is like making all users in the group into Dataset Customers.

iTHRIV COMMONS OBJECT LEVEL VIEW ACCESS FLAGS

Access to view project metadata, dataset metadata and dataset files are controlled by Project Owners and Dataset Admins (respectively) using object level flags as described below.

 1.       PROJECT METADATA ACCESS FLAGS:

Project metadata access flag can be either ‘private’ or ‘public’. By default when a project is created the metadata is set to private. A project owner can only change this flag. When metadata is private only project owners and collaborators can view the metadata. Anyone can view project metadata when it is set to public. If the Project Owner sets the project metadata access to Public, the summary information about the project is visible to users outside of the Project team (even without logging in to the system).

 2.       DATASET METADATA ACCESS FLAGS:

Dataset metadata access flags can be either ‘private’ or ‘public’. By default when a dataset is created its metadata is set to private. Dataset admin can only change this flag. When dataset metadata is private, only project owners, collaborators and dataset admins can see the metadata. If the Dataset Admin sets the dataset metadata access to Public, the summary information about the dataset is visible to users outside of the Project team (even without logging in to the system).  This is NOT the same as getting access to the dataset itself.

 3.       DATASET DOWNLOAD ACCESS FLAGS:

Dataset access flags can be either ‘private’ or ‘public’. This access flag can be set only by the dataset admin. When dataset is private, only dataset customers, dataset collaborators, and dataset admins can download the dataset. The interface will not allow this to be set to ‘public’ unless appropriate institutional requirements have been met.  It is the responsibility of the dataset admin to truthfully and accurately complete the metadata fields regarding HIPAA.  When a dataset is public it can be downloaded by any logged in user and any public user (no login).  Setting this flag to PUBLIC is like making ALL users into Dataset Customers.  Note that only the CURRENT version of the file will be visible and available for download for the public.  Also, the dataset metadata must first be made public for the dataset to be made public.

# iTHRIV Commons Project and Dataset Metadata

This section describes all the metadata (required metadata and optional metadata) that the iTHRIV Research Data Commons stores for project and datasets. Any reference to the ‘user’ in this document is in the context of an authorized and authenticated user of the system.

## Project Metadata

Metadata that needs to be stored for any project is described in this section. Where appropriate we are using metadata terms as per the schema at <http://schema.org>. Some of the metadata is created by the system and some needs to be provided by the user. Some of the metadata is required and some are optional as indicated for each of the metadata terms/fields listed below:

- **@context**: (*required, system generated, and immutable*). This field always has the value: <http://schema.org>
- **@type**: (*required, system generated, and immutable*). This field always has the value: CreativeWork. This is a schema.org metadata term (Schema.Thing.CreativeWork).
- **projectPI** (required, user provides one or more values and can be updated by the user). This field is used for storing the email of one or more project principal investigators. Note that any new project pi added as part of this metadata field gets the project owner role on the project. However, deleting any project pi as part of this metadata field doesn’t remove the corresponding project owner role. This is NOT a schema.org metadata term. This field has the following two sub fields:
  - @type: (required and system generated). The value for the field is always: Person
  - email: (required and user provided). Email of the project principal inverstigator.
- **user**: (required, user provides one or more values and can be updated by the user): This field is used for storing user access permission on a project. This is NOT a schema.org metadata term. This field has the following three sub fields:
  - @type: (required and system generated). The value for the field is always: Person
  - email: (required and user provided). Email of the user who has access to the project.
  - roleID: (required and user provided and can be updated by the user). The role id is an integer that represents a specific project role (owner or collaborator). This field is for application internal use only.

**Note**: This metadata field is used for project access permission.

- **identifier**: (*required, system generated, and immutable*). This field is used for storing the ID for a project which uniquely identifies a project in the system. This is a schema.org metadata term (Schema.Thing.identifier).
- **name**: (*required, user provides a value and can be updated by user*): This field is used for storing the name or title of the project. This is a schema.org metadata term (Schema.Thing.name).
- **description**: (*required, user provides a value and can be updated by user*): This term is used for describing the project. This is a schema.org metadata term (Schema.Thing.description).
- **keywords**: (*required, user provides one or more values and can be updated by user*): This field is used for storing keywords or tags used for describing the project. This is a schema.org metadata term (Schema.Thing.CreativeWork.keywords).
- **alternateName**: (*optional, user provides a value and can be updated by user*): This field is used for storing another (alias) name for the project. This is a schema.org metadata term (Schema.Thing.alternateName).
- **dateCreated**: (*required, system generated and immutable*): This field is used for storing the date-time when the project gets created in the system. This is a schema.org metadata term (Schema.Thing.CreativeWork.dateCreated).
- **dateModified**: (*required and system generated*): This field is used for storing the date-time when the project gets updated in the system. This is a schema.org metadata term (Schema.Thing.CreativeWork.dateModified).
- **contactPerson**: (optional, user provides one or more values and can be updated by the user). This field is used for storing the email of one or more project team members. Note that the primary project user roles are stored elsewhere and not part of the project metadata. This is NOT a schema.org metadata term. This field as the following two sub fields:
  - @type: (required and system generated). The value for the field is always: Person
  - Email: (required and user provided). Email of the project team member.
- **hostOrganization**: (required, system generated and immutable). This is the host organization where the project metadata gets stored when it is created in the system. In a federated system of data storage, a client can use this field to know where the metadata is stored for a project. This is a NOT a schema.org metadata term.
- **sourceOrganization**: (*required, user provides a value and can be updated by the user*). This field is used to store the name of the organization to which the project belongs. This is a schema.org metadata term (Schema.Thing.CreativeWork.sourceOrganization). This field has the following two sub-fields:
  - **@type**: (*required and system generated*). The value for this field is always: Organization
  - **name**: (*required, user provides a value and can be updated by the user*). Name of the organization
- **isMetadataPublic**: (*required, user provides the value, and can be updated by the user*). This field is used to indicate if the project is ‘FALSE or ‘TRUE. When a project gets created, the system assigns ‘FALSE’ by default. When a project has ‘FALSE’ status the project metadata is accessible only to those who have been given access to the project. Metadata for PUBLIC projects are accessible to anyone (no access permission is needed). See ‘Data Commons Access Policy’ document for details. This is NOT a schema.org metadata term.

**Note**: This metadata field is used for project metadata visibility

- **funder**: (*optional, user provides one or more values and can be updated by the user*): This field is used for storing one or more organizations that support a project. This is a schema.org metadata term (Schema.Thing.CreativeWork.funder). There can be more than one organization as sponsor for a given project. This field has the following two sub-fields:
  - **@type**: (*required and system generated*). The value for this field is always: Organization
  - **name**: (*required, user provides the value, and can be updated by the user*). This field is used for storing the name of the organization.
- **partner**: (*optional, user provides one or more values and can be updated by the user*): This field is used for storing the name of an organization that has a partner role in the project. This is NOT a schema.org metadata term. There can be more than one organization as partner for a given project. This field has the following two sub-fields:
  - **@type**: (*required and system generated*). The value for this field is always: Organization
  - **name**: (*required, user provides a value and can be updated by the user*). This field is used for storing the name of the partner organization.
- **webPageUrl**: (*optional, user provides one or more values and can be updated by the user*): This field is used for storing the url(s) for any number of project related webpages/web resources. This is NOT a schema.org metadata term.
- **portalResourcesUrl**: (*optional, user provides one or more values and can be updated by the user*): This field is used for storing url(s) for iTHRIV Research Concierge Portal resources and events that Research Commons project owners choose to associate with their project.  These will typically represent resources the project team plans to use and indexing them in the project will help planning. This is NOT a schema.org metadata term.
- **document**: (optional, system generated and updated by the system). This field is used to store information about the files uploaded to a project. These files are contract, IRB approval and other administrative type documents. This field can be used to store one or more files uploaded to the project. This is NOT a schema.org metadata term. This field has the following sub-fields:
  - fileName: This field is used to store the name of the file.
  - fileSize: This field is used to store the size (in bytes) of the file.
  - category: This field is used to store the type of document (e.g., Contract, IRB Approval, etc.)
  - dateCreated: This field is used to store the date on which the file is uploaded to the project.
  - fileVersion: This field is used for storing the version of the file.
  - encodingFormat: This field is used for storing the MIME type of the file.
  - fileCreatedBy: This field is used for storing the email of the user who uploaded the file.
  - url: This field is used to store the url path of the file.

## Generic Dataset Metadata

Metadata that needs to be stored for any generic dataset is described in this section. Where appropriate we are using metadata terms as per the schema at <http://schema.org>. Some of the metadata is created by the system and some needs to be provided by the user. Some of the metadata is required and some are optional as indicated for each of the metadata terms/fields listed below:

- **@context**: (required, *system generated, and immutable*). This term always has the value: <http://schema.org>
- **@type**: (required, *system generated, and immutable*). This term always has the value: Dataset. This is a schema.org metadata term (Schema.Thing.CreativeWork.dataset)
- **identifier**: (*required, system generated, and immutable*). This field is used for storing the ID for a dataset which uniquely identifies a dataset in the system. This is a schema.org metadata term (Schema.Thing.identifier).
- **name**: (*required, user provides a value and can be updated by user*): The field is used for storing the name or title of the dataset. This is a schema.org metadata term (Schema.Thing.name).
- **description**: (*required, user provides a value and can be updated by user*): This field is used for describing the dataset. This is a schema.org metadata term (Schema.Thing.description).
- **keywords**: (*required, user provides one or more values and can be updated by user*): This field is used for storing keywords or tags used for describing the dataset. This is a schema.org metadata term (Schema.Thing.CreativeWork.keywords).
- **url**: (*required and system generated*). This field is used for storing the url path of the data file for data file downloading. This is a schema.org metadata term (Schema.Thing.url). Note: A value for this field is required only when the dataset contains a data file.
- **fileSize**: (*required and system generated*). This field is for storing the size (in bytes) of the data file. This is schema.org metadata term (Schema.Thing.fileSize). Note: A value for this field is required only when the dataset contains a data file.
- **fileName**: (*required and system generated*). This field is for storing the name of the data file. This is NOT a schema.org metadata term. Note: A value for this field is required only when the dataset contains a data file.
- **encodingFormat**: (*required and system generated*). This field is for storing the MIME type of the of the data file. This is schema.org metadata term (Schema.Thing.encodingFormat). Note: A value for this field is required only when the dataset contains a data file.
- **fileDateCreated**: (*required and system generated*). This field is for storing the date of uploading a file to a dataset. This is NOT a schema.org metadata term. Note: A value for this field is required only when the dataset contains a data file.
- **fileVersion**: (*required and system generated*). This field is for storing latest version number for the file uploaded to a dataset. A dataset can have more than one version of a data file. This is NOT a schema.org metadata term. Note: A value for this field will exist only when the dataset has a current version of a data file.
- **fileChecksum**: (*required and system generated*). This field is for storing checksum value (md5) of the file uploaded to a dataset. The checksum value is for data integrity check. This is NOT a schema.org metadata term. Note: A value for this field will exist only when the dataset has a data file.
- **fileCreatedBy**: (*required and system assigned*). This field is used to store the email of the user who uploaded the file to a dataset. This is NOT a schema.org metadata term. Note: A value for this field will exist only when the dataset has a current version of a data file.
- **fileHIPAAWarning**: (*system generated for De-ID/LDS dataset*): This field is used to store HIPAA identifiers warnings (e.g., PossibleDateData, PossibleZipData etc) if the system finds HIPAA identifies (Date, Zip code, SSN, MRN, Phone, Email) in the uploaded data file for a De-ID/LDS dataset. This is NOT a schema.org metadata term.
- **dateCreated**: (*required, system generated and immutable*): This field is used for storing the date/time at which the dataset metadata got created in the system. This is a schema.org metadata term (Schema.Thing.CreativeWork.dateCreated).
- **dateModified**: (*required and system generated*): This field is for storing the date/time at which the dataset metadata got updated in the system. This is a schema.org metadata term (Schema.Thing.CreativeWork.dateModified).
- **isMetadataPublic**: (*required, user provides the value, and can be updated by the user*). This field is used to indicate if the dataset metadata is ‘FALSE’ or ‘TRUE’. When a dataset gets created, the system assigns ‘FALSE’ by default. When this field is set to ‘FALSE the dataset metadata is accessible only to those who have been given access to the dataset. This is NOT a schema.org metadata term (previously created for project metadata).

**Note**: This metadata field is used for dataset metadata access permission. When this field is set to ‘TRUE’ metadata is accessible to anyone (no access permission is needed). See ‘Data Commons Access Policy’ document for details.

- **isDataPublic**: (*required, user provides the value, and can be updated by the user*). This field is used to indicate if the dataset data is ‘FALSE’ or ‘TRUE’. When a dataset gets created, the system assigns ‘FALSE’ by default. When this field is set to ‘TRUE the dataset data is accessible (can download the data file) only to those who have been given access to the dataset. This is NOT a schema.org metadata term.

**Note**: This metadata field is used for dataset data access permission. When this field is set to ‘TRUE’ current version of the dataset is available for download only to anyone (no access permission is needed). See ‘Data Commons Access Policy’ document for details.

- **user**: (*required, user provides one or more values and can be updated by the user*): This field is used for storing user access permission on a dataset. This is NOT a schema.org metadata term. This field has the following three sub fields:
  - @type: (required and system generated). The value for the field is always: Person
  - email: (required and user provided). Email of the user who has access to dataset.
  - roleID: (required and user provided and can be updated by the user). The role id is an integer that represents a specific dataset role (dataset administrator, dataset collaborator, dataset customer or dataset customer group). This field is for application internal use only.

**Note:** This metadata field is used for dataset access permission.

- **sourceProject**: (required, system generated and immutable): This field is used for associating the project in which the dataset was originally created. The ID of the project is stored in this field. This is NOT a schema.org metadata term.
- **isPartOf**: (*required, user provides one or more values for this field and can be updated by the user*): This field is used for associating a dataset with a project. The ID of the associated project is stored in this field. A dataset can be associated with more than one project. This a schema.org metadata term (Schema.Thing.CreativeWork.isPartOf).
- **isBasedOn**: (optional*, user provides one or more values for this field and can be updated by the user*): This field is used for associating the dataset with another dataset if the dataset has been derived from that dataset. The ID of the dataset from which this dataset has been derived is stored in this field. A dataset can be derived from more than one dataset. This a schema.org metadata term (Schema.Thing.CreativeWork.isBasedOn).
- **hostOrganization**: (required, system generated and immutable). This is the host organization where the dataset gets stored when it is created in the system. In a federated system of data storage, a client can use this field to know where the metadata and data is stored for a dataset. This is a NOT a schema.org metadata term.
- **sourceOrganization** : (*required, user provides a value and can be updated by the user*). This field is used to store the name of the organization to which the creator of the dataset belongs. This is the host organization where the dataset gets stored when it is created in the system. This is a schema.org metadata term (Schema.Thing.CreativeWork.sourceOrganization). This field has the following two sub-fields:
  - **@type**: (*required and system generated*). The value for this field is always: Organization
  - **name**: (*required, user provides a value and can be updated by the user*). Name of the organization
- **identifiersHIPAA**: (*required, user provides one or more personal identifiable as defined in HIPPA, and can be updated by the user*). This field is used to store one or more HIPAA related identifiers that the dataset may contain. This is NOT a schema.org metadata term.
- **otherSensitiveData**: (*required, user provides one or more values and can be updated by the user*). This field is used to store one or more values that are considered as sensitive data types that the dataset may contain. For information on sensitive data, refer to <https://security.virginia.edu/university-data-protection-standards>. This is NOT a schema.org metadata term.
- **sensitivityLevel**: (*required, system sets the value and can’t be updated by the user*). This field is used to store dataset sensitivity level – one of the 3 possible values (HIGH SENSITIVITY DATA, LIMITED DATASET, DE-IDENTIFIED). System sets this value based on the data stored in the metadata field ‘**identifiersHIPAA’**. This is NOT a schema.org metadata term.
- **studyIRBNumber**: (required for HSD dataset, user provides a value for this field and can be updated by the user). This field is used for storing the IRB number of the study that the dataset is part of. This is NOT a schema.org metadata term.
- **variableMeasured**: (*optional, user provides the name of one or more variables used in dataset, and can be updated by the user*). This field is used for storing the name of the variable measured to generate data in the dataset. This is a schema.org metadata term (Schema.CreativeWork.Dataset.variableMeasured).
- **approvedIRB**: (*optional, user provides a value for this field and can be updated by the user*). This field is used for associating a project document (a file) that is already in the project as the IRB approval document for the dataset. This is NOT a shema.org metadata term.
- **contract**: (*optional, user provides a value for this field and can be updated by the user*). This field is used for associating a project document(s) that is already in the project as the contract document for the dataset. This is NOT a shema.org metadata term.
- **license**: (*optional, user provides a value for this field and can be updated by the user*). This field is used for specifying any license requirement for using the dataset. This is a schema.org metadata term (Schema.CreativeWork.license).
- **spatialCoverage**: (*optional, user provides one or more values for this field and user can update this field*). This field is used for specifying location (place) to which the dataset is related. This is a schema.org metadata term (Schema.CreativeWork.spatialCoverage). This field has the following two sub-fields:
  - **@type**: (*required and system generated*). The value for this field is always: Place
  - **address**: (*required, user provides a value and can be updated by the user*). Name of the place to which the dataset is related.
- **temporalCoverage**: (*optional, user provides one value for this field and user can update this field*). This field is used for specifying the time period for which the dataset has been collected. This is a schema.org metadata term (Schema.CreativeWork.temporalCoverage). This field has the following three sub-fields:
  - **@type**: (*required and system generated*). The value for this field is always: DateTime
  - **startDate**: (*required and user provides a value*). Start date of the time period in which the data for the dataset has been collected.
  - **endDate**: (*optional and user provides a value*). End date of the time period in which the data for the dataset has been collected.
- **linkToExternalDataset**: (*optional, user provides a link to a dataset; can be updated by the user*). This field is used for storing the URL of an external dataset (data lives outside of the data commons system) or an internal research database (e.g. REDCap). This field is NOT a schema.org metadata term. A value for this field will be allowed only if the dataset does not have data file; similarly, if this field is populated, the user will not have the option to upload a file.

- **publisher**: (required*, system generated based on where dataset is hosted at the time of making the dataset public – one of the partner institutions name)* This is schema.org metadata term (Schema.Thing.publisher)
- **associatedDSP**: (*required for HSD dataset and user provides a value*): This field is used for storing the url of the Data Security Plan (DSP) document of the parent project. A value for this field is required when creating a HSD dataset. This field is NOT a schema.org metadata term.
- **consentRestrictions**: (*optional, user provides a value for any use restrictions placed on the data through participant informed consent. Applies only to human subjects research).* This is NOT a shema.org metadata term. The value for this field may be ***Limited to study team; Limited to a single university; Data use limit (timeframe for use); Minors (reconsent needed);*** or ***Reuse for another study.*** There is no default value for this field.

**NOTE*:*** This metadata field will be supported in a future version of Commons.

- **retentionDuration**: (*required prior to making dataset data file public, user provides the retention period for the dataset*). This field is used for storing the retention period of the dataset. The value for this field may be ***Indefinitely***; ***Must be destroyed after publication is complete***; ***Minimum of 6 years*** (for datasets containing PHI); ***IND/IDE retention***; or ***Must receive sponsor permission before destruction***. This is NOT a schema.org metadata term.

**NOTE*:*** This metadata field will be supported in a future version of Commons.

- **retentionDestructionDate**: (*optional, user provides a value for this field*) This field stores planned destruction date when known. This is NOT a schema.org metadata term.

**NOTE*:*** This metadata field will be supported in a future version of Commons.

- **retentionComment**: (*optional, user provides a value for this field*). This field is used to store clarification on the dataset retention period. This is NOT a schema.org metadata term.

**NOTE*:*** This metadata field will be supported in a future version of Commons.

## REDCap Dataset Metadata

REDCap metadata includes all the metadata defined earlier for the Generic Dataset. Metadata that is specific to dataset objects of type REDCap is described in this section. Automated, scheduled data extracts from REDCap are optional for all REDCap projects. The user can elect to set up an auto-extract of data from a REDCap project report. They request the extract via the user-interface, at which point the associated report ID and refresh rate must be specified. Once the report extract is set up by REDCap staff, the other system fields listed below will auto-populate. Where appropriate we are using metadata terms as per the schema at <http://schema.org>.

- **redCapProjectURL**: (required, user provides a value for this field*, and can be updated by the user*). This field is used to store the URL of a specific REDCap project. This field is NOT a schema.org metadata term.
- **redCapExtractData**: (required, user provides a value for this field*, and can be updated by the user*). This field is used to store a True/False value reflecting whether or not an auto-extract (cvs file exported from REDCap to Commons) is associated with this dataset object.
- **redCapReportID**: (optional, user provides a value for this field*, and can be updated by the user*). This field is used to store the ID of a report of a REDCap project. The user specified report id used for extracting a limited set of data from the REDCap project. If the user didn’t set a value for this field and redCapExtractData==TRUE then all data of a REDCap project will be extracted. This field is NOT a schema.org metadata term.
- **redCapRefreshRate**: (required IF redCapExtractData=TRUE, user provides a value for this field*, and can be updated by the user*). This field is used to store time interval (as number of days) in which data needs to be retrieved from REDCap server and updated in commons. This field is NOT a schema.org metadata term.
- **redCapProjectTitle**: (system generated IF redCapExtractData=TRUE). This field is used to reflect the title of the REDCap project as listed in REDCap at the time of an auto-extract from REDCap to the Commons. Edits to this fields must be made in REDCap. This field is NOT a schema.org metadata term.
- **redCapProjectPI**: (system generated IF redCapExtractData=TRUE). This field is used to reflect the name of the REDCap project PI as listed in REDCap at the time of an auto-extract from REDCap to the Commons. Edits to this fields must be made in REDCap. This field is NOT a schema.org metadata term. This field has the following two sub-fields:
  - **@type**: (*required and system generated*). The value for this field is always: Person
  - **name**: (*required, user provides a value and can be updated by the user*). Name of the PI.

## DICOM Dataset Metadata

DICOM metadata includes all the metadata defined earlier for the Generic Dataset. Metadata that is specific to dataset objects of type DICOM is described in this section.

- **deIdentified**: (*required, system generated and* *can’t be updated by the user*). This field is used to store True/False value to indicate if the DICOM study has been de-identified or not. This field will be set as part of pre-processing script that processes the DICOM files. This field is NOT a schema.org metadata term.
- **bidsStructure**: (*required, system generated, and can’t be updated by the user*). This field is used to store True/False value to indicate if the DICOM study files are in BIDS structure or not. This field will be set as part of pre-processing script that processes the DICOM files. This field is NOT a schema.org metadata term.
- **quality**: (*required, system generated, and can’t be updated by the user*). This field is used to store one of the values (Unknown, Good, Medium, Poor) to indicate the quality of the DICOM image. This field will be set as part of pre-processing script that processes the DICOM files. This field is NOT a schema.org metadata term.
- **studyDate**: (*required, system generated* *, and can’t be updated by the user*). This field is used to store the date on which the study took place. This field will be set as part metadata extraction from DICOM files. This field is NOT a schema.org metadata term.
- **scannerManufacturerName**: (*required, system generated, and can’t be updated by the user*). This field is used to store the name of the manufacturer of scanner used in scanning the subject. . This field will be set as part metadata extraction from DICOM files. This field is NOT a schema.org metadata term.
- **scannerModelName**: (*required, system generated, and can’t be updated by the user*). This field is used to store the model name of the scanner used in scanning the subject. . This field will be set as part metadata extraction from DICOM files. This field is NOT a schema.org metadata term.
- **organName**: (*required, system generated, and can’t be updated by the user*). This field is used to store the name of the body part that is part of the study (body part for which the DICOM images have been taken). This field will be set as part metadata extraction from DICOM files. This field is NOT a schema.org metadata term.
- **fieldOfView**: (*required, system generated, and can’t be updated by the user*). This field is used to store angular measurement of field of view of the organ being scanned (a numeric value). This field will be set as part metadata extraction from DICOM files. This field is NOT a schema.org metadata term.
- **fieldStrength:** (*required, system generated, and can’t be updated by the user*). This field is used to store the magnetic field strength used in scanning (a numeric value). This field will be set as part metadata extraction from DICOM files. This field is NOT a schema.org metadata term.
